# Cerebrospinal fluid CXCL13 in Lyme neuroborreliosis and asymptomatic HIV infection

**DOI:** 10.1186/1471-2377-13-2

**Published:** 2013-01-07

**Authors:** Daniel Bremell, Niklas Mattsson, Mikael Edsbagge, Kaj Blennow, Ulf Andreasson, Carsten Wikkelsö, Henrik Zetterberg, Lars Hagberg

**Affiliations:** 1 Department of Infectious Medicine, Institute of Biomedicine, the Sahlgrenska Academy, University of Gothenburg, Gothenburg, Sweden; 2 Clinical Neurochemistry Laboratory, Institute of Neuroscience and Physiology, Department of Psychiatry and Neurochemistry, the Sahlgrenska Academy, University of Gothenburg, Gothenburg and Mölndal, Sweden; 3 Hydrocephalus Research Unit, Institute of Neuroscience and Physiology, Department of Clinical Neuroscience and Rehabilitation, the Sahlgrenska Academy, University of Gothenburg, Gothenburg, Sweden; 4 UCL Institute of Neurology, Queen Square, London, WC1N 3BG, UK

## Abstract

**Background:**

It has been suggested that cerebrospinal fluid (CSF) CXCL13 is a diagnostic marker of Lyme neuroborreliosis (LNB), as its levels have been shown to be significantly higher in LNB than in several other CNS infections. Levels have also been shown to decline after treatment with intravenous ceftriaxone, but levels after treatment with oral doxycycline have previously not been studied. Like *Borrelia burgdorferi*, HIV also has neurotropic properties. Elevated serum CXCL13 concentrations have been reported in HIV patients, but data on CSF levels are limited.

**Methods:**

We longitudinally analysed CSF CXCL13 concentrations in 25 LNB patients before and after oral doxycycline treatment. Furthermore, we analysed CSF CXCL13 concentrations in 16 untreated LNB patients, 27 asymptomatic untreated HIV-1 infected patients and 39 controls with no signs of infectious or inflammatory disease.

**Results:**

In the longitudinal LNB study, initially high CSF CXCL13 levels declined significantly after doxycycline treatment, which correlated to a decreased CSF mononuclear cell count. In the cross-sectional study, all the LNB patients had CSF CXCL13 levels elevated above the lowest standard point of the assay (7.8 pg/mL), with a median concentration of 500 pg/mL (range 34–11,678). Of the HIV patients, 52% had elevated CSF CXCL13 levels (median 10 pg/mL, range 0–498). There was a clear overlap in CSF CXCL13 concentrations between LNB patients and asymptomatic HIV patients. All but one of the 39 controls had CSF CXCL13 levels below 7.8 pg/mL.

**Conclusions:**

We confirm previous reports of highly elevated CSF CXCL13 levels in LNB patients and that these levels decline after oral doxycycline treatment. The same pattern is seen for CSF mononuclear cells. CSF CXCL13 levels are elevated in neurologically asymptomatic HIV patients and the levels overlap those of LNB patients. The diagnostic value of CSF CXCL13 in LNB remains to be established.

## Background

Lyme neuroborreliosis (LNB) is the most common arthropod-borne CNS infection in Europe and the USA [1]. The diagnosis of LNB rests on a combination of anamnestic, clinical and laboratory findings. For a definite diagnosis, European guidelines require clinical symptoms consistent with LNB, such as painful meningoradiculitis, other diagnoses excluded, cerebrospinal fluid (CSF) pleocytosis and intrathecal *Borrelia burgdorferi* (*Bb*) antibody production [2,3]. There are some limitations to this diagnostic procedure. CSF pleocytosis is not specific to LNB but is seen in other infectious and non-infectious diseases of the central nervous system. Intrathecal antibody production may be absent for up to six weeks after the onset of symptoms and intrathecally produced antibodies are known to remain positive for years after the initial disease [4,5].

In recent years, the chemokine CXCL13 (also known as BLC – B lymphocyte chemoattractant) in CSF has been presented as a potential marker of LNB, more specific than today’s diagnostic approach [6]. CXCL13 is a potent attractor of B cells and CSF levels rise early in the course of LNB and decline after treatment [7]. Significantly higher CSF CXCL13 concentrations are recorded in LNB than in several other infectious and inflammatory CNS diseases [6-8]. Cryptococcosis and neurosyphilis are among the few other infectious diseases in which CSF CXCL13 levels as high as those in LNB have been reported [9,10].

Oral doxycycline and intravenous ceftriaxone are considered equally effective for the treatment of LNB with peripheral nervous system symptoms, while intravenous ceftriaxone is still the preferred treatment of LNB with central nervous system symptoms such as meningitis or encephalitis in many centra [2].

HIV infects the central nervous system at the primary infection or shortly thereafter, causing a chronic, low-grade inflammatory reaction in most clinically asymptomatic patients [11,12]. In HIV patients, serum levels of CXCL13 have been shown to be significantly elevated when compared with controls [13]. van Burgel et al. described elevated CSF levels of CXCL13 in six patients with HIV meningitis but low levels in seven patients with HIV infection without intrathecal inflammation [10]. CSF levels of CXCL13 in a larger group of patients with asymptomatic HIV infection have not been studied.

We analysed CSF levels of CXCL13 in patients with LNB, HIV and controls, in order to assess whether the test is specific for LNB even when compared with patients with HIV infection. We have also studied how doxycycline treatment of LNB affects CSF CXCL13 levels and the CSF cell count by analysing a well-characterised set of LNB patients before and after treatment.

## Methods

### Study subjects

We enrolled different study participants for the longitudinal and the cross-sectional study. For the longitudinal study, we analysed 25 LNB patients who had undergone CSF sampling before and after treatment between October 1995 and November 2005. All these patients were treated with 200–400 mg of oral doxycycline daily for 10–14 days, according to Swedish recommendations [14]. For the cross-sectional study, we analysed three groups of patients: patients with LNB, patients with HIV-1 infection and controls with no infectious or inflammatory disease. The inclusion criteria for LNB patients in the cross-sectional part of the study were LNB diagnosed between January 2005 and June 2009 and CSF sampling before the start of treatment. The inclusion criteria for HIV patients were: I. Asymptomatic infection; II. No antiretroviral treatment; III. No clinical signs of neurological disease; IV. A negative test for syphilis. CSF sampling of the HIV patients was undertaken between January 2005 and June 2009. The control group included two subgroups. One subgroup consisted of 18 patients with neurological symptoms such as headache, vertigo and radiculitic pain, where an underlying organic neurological disease had been ruled out and where CSF sampling had been undertaken as part of the investigations. The other subgroup consisted of 21 subjects with no neurological disease or symptoms who had undergone lumbar puncture for CSF sampling as part of a research project unrelated to this study [15]. In accordance with Swedish regulations, informed consent was obtained from all participants prior to inclusion of their CSF and blood samples into the biobank from which the samples then were retrieved. The study was approved by the regional ethical review board at the University of Gothenburg.

### LNB diagnostic criteria

The diagnostic criteria for LNB were: I. Symptoms consistent with LNB and other diagnoses excluded; II. CSF mononuclear pleocytosis with a mononuclear cell count of > 5/μL and erythrocytes of < 100/μL; III. *Bb*-specific antibodies in CSF above the upper reference level; IV. At least one of the following: a) a positive antibody index (AI), defined as the ratio of the CSF/serum quotient of specific *Bb* antibodies to the corresponding CSF/serum quotient of total immunoglobulins, where AI values of ≥ 1.5 are considered positive, b) CSF cytological examination consistent with LNB with activated plasma cells [16].

### CSF sampling

All CSF samples were collected by lumbar puncture in the L3/L4 or the L4/L5 inter-space. The first 5–12 mL of CSF were collected in a polypropylene tube and immediately transported to the local laboratory for centrifugation. The supernatant was pipetted off, gently mixed to avoid possible gradient effects and aliquoted in polypropylene tubes that were stored at −80°C pending biochemical analyses, without being thawed and re-frozen.

### Biochemical analyses

CXCL13 was measured by ELISA (Human CXCL13/BLC/BCA-1 Immunoassay, R&D Systems Inc., Abingdon, United Kingdom), according to instructions from the manufacturer. Based on measurements of duplicates of the standard samples (concentrations 7.8-500 mg/L), the average intra-assay CVs were ≤ 10%. Syphilis testing was done with LIAISON Treponema Screen (Diasorin, Saluggia, Italy). For the analysis of *Bb* antibodies in serum and CSF, two different tests were used during the study period. Until 26 June 2006, antibodies were analysed using an enzyme-linked immunosorbent assay (ELISA) kit for IgG and IgM antibodies (Dako Lyme Borreliosis Kit, Dako Cytomation, Glostrup, Denmark). Tests positive for IgM were further analysed with a more specific test (IDEIA, Dako Cytomation, Glostrup, Denmark). After 26 June 2006, *Bb* antibodies were analysed using a sandwich chemiluminescence immunoassay (CLIA) test kit (Diasorin, Saluggia, Italy). HIV RNA in serum and CSF was determined using a quantitative polymerase chain reaction (Amplicor, HIV-1 Monitor Test version 1.5, Roche Diagnostic Systems, Hoffman-La Roche, Basel, Switzerland).

### Statistics

Statistical analysis was performed using GraphPad Prism 5.0 (GraphPad Software, San Diego, USA). Data are presented as the median (range). CXCL13 values below the detection limit of 7.8 ng/mL were assigned a value of 3.9 ng/mL for graphical purposes. Analyses were made using non-parametric methods. In the longitudinal study, differences before and after treatment were analysed with the Wilcoxon matched pairs test. Differences between groups in the cross-sectional study were analysed with the Kruskal-Wallis test followed by Dunn’s post test. Correlations were analysed using the Spearman rank order correlation. P values of < 0.05 were considered significant.

## Results

**Figure 1:**
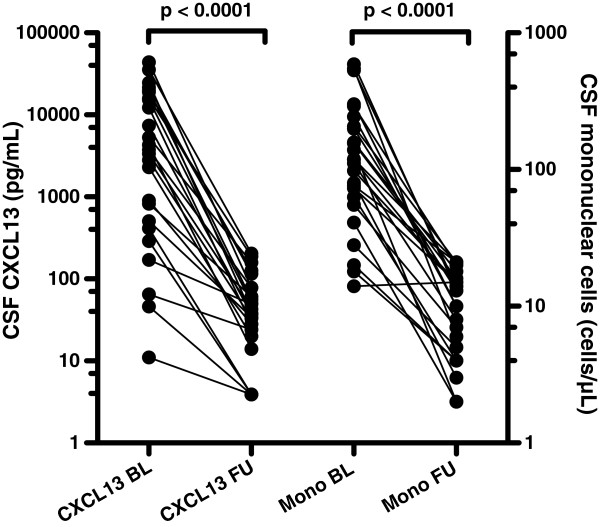
**CSF levels of CXCL13 and mononuclear cells before and after treatment of Lyme neuroborreliosis.** Pairwise comparisons of CXCL13 and mononuclear cells in cerebrospinal fluid before and after treatment of Lyme neuroborreliosis. P-values from the Wilcoxon matched pairs test. BL – baseline, FU – follow-up, Mono – mononuclear cells. Median time between CSF samplings was 45 days (range 33–75).

**Figure 2:**
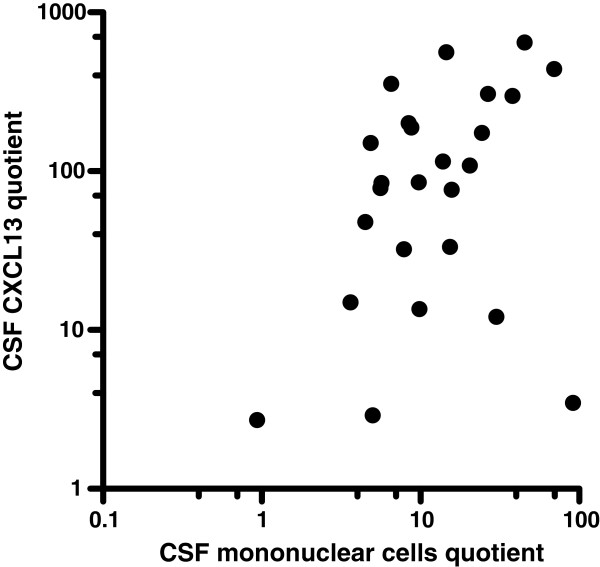
**Quotients of CSF mononuclear cells and CSF CXCL13 before and after treatment.** Quotients are calculated as (CSF mononuclear cells before treatment)/(CSF mononuclear cells after treatment) and (CSF CXCL13 before treatment)/(CSF CXCL13 after treatment). Spearman *r* = 0.37, P = 0.036.

In the longitudinal part of the study, 25 LNB patients were analysed. Baseline data, clinical symptoms and routine CSF analyses are shown in Table 1. 23 of the patients had a positive AI. One patient had an AI of 1.1 and for one patient the AI could not be calculated due lack of data for total immunoglobulins. The latter two patients had a CSF cytological examination consistent with LNB with activated plasma cells. The median time between CSF samplings was 45 days (33–75). Before treatment, the median CSF CXCL13 was 3,727 pg/mL (range 11–43,746), >which declined after treatment to 38 pg/mL (3.9-204) (P < 0.001) (Figure 1). The decline in the CSF mononuclear cell count after treatment was also significant: median 118 cells/μL (14–590) before treatment, versus a median of 13 cells/μL (2–21) after treatment, P < 0.001 (Table 1 and Figure 1). The quotients before and after treatment of CSF CXCL13 and CSF mononuclear cells were calculated as (CSF CXCL13 before treatment)/(CSF CXCL13 after treatment) and (CSF mononuclear cell before treatment)/(CSF mononuclear cells after treatment). The quotients correlated significantly (Spearman r = 0.37, P = 0.036) (Figure 2).

**Table 1:** Baseline data, symptoms and routine CSF analyses

	**Longitudinal study**		**Cross-sectional study**		
	**LNB**		**LNB**	**HIV**	**Controls**
**N**	25		16	27	39
**M/F**	17/8		7/9	16/11	17/22
**Age (years)**	50		37	38	64
	(12–74)		(25–67)	(23–76)	(27–76)
**Duration of symptoms (days)**	28		21	na	na
	(5–360)		(7–120)		
**Symptoms**					
radiculitis	15		10	na	na
headache			4	na	na
facial palsy	3			na	na
radiculitis and facial palsy			1	na	na
radiculitis and other palsy	2		1	na	na
radiculits and sensibility disturbancies	3			na	na
other palsy	2			na	na
	**Baseline**	**Follow-up**			
**CSF mononuclear cells (cells/μL)**	118	13	58	4	1
	(14–590)	(2–21)	(8–493)	(0–69)	(0–8)
**CSF albumin (mg/L)**	664	301	432	203	230
	(267–2108)	(146–707)	(206–1080)	(108–383)	(43–465)
**Blood CD4 cells (cells/μL)**	na		na	250	na
				(20–940)	
**Blood viral count (copies/μL)**	na		na	54500	na
				(2920–450000)	
**CSF viral count (copies/μL)**	na		na	5680	na
				(0–384000)	

Baseline data, clinical symptoms and routine CSF analyses for the longitudinal and the cross-sectional study. Data are presented as median (range). LNB – Lyme neuroborrelios, na – not applicable, other palsy – palsy other than facial palsy.

In the cross-sectional part of the study, 85 patients were analysed; 16 with LNB, 27 with HIV infection and 39 controls without inflammatory CNS disease. Baseline data, clinical symptoms and routine CSF analyses are shown in Table 1. All 16 LNB patients had a positive AI indicating intrathecal antibody production. For LNB patients, the median duration of neurological symptoms was 21 days (7–120). For HIV patients, the median time since diagnosis was 15 months (1–180). There was no significant difference in age between patients with LNB and HIV infection (median 37 and 38 years respectively), while the controls were significantly older, with a median age of 64 years (P < 0.01). CSF levels of mononuclear cells differed significantly between all three groups; they were highest in LNB patients, with a median of 58 cells/μL (8–493), followed by HIV patients at 4 cells/μL (0–69) and controls at 1 cell/μL (0–8) (P < 0.01).

**Figure 3:**
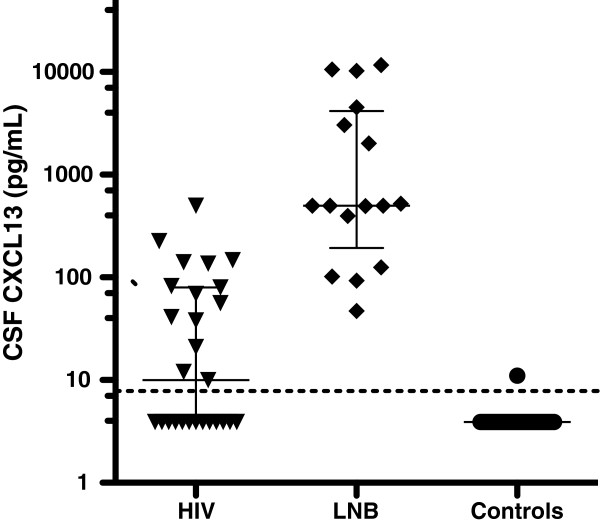
**CSF levels of CXCL13 in patients with Lyme neuroborreliosis, HIV infection and controls.** CSF CXCL13 levels in untreated, asymptomatic HIV patients, untreated LNB patients and controls. Lines showing median and interquartile range for each group. Dotted line at the lowest standard point of the assay (7.8 pg/mL). Values below the detection limit were assigned a value of 3.9 pg/mL for graphical purposes. LNB – Lyme neuroborreliosis.

CSF CXCL13 levels differed significantly between all three groups of patients in the cross-sectional study (Figure 3) (P < 0.01). All LNB patients had concentrations above the lowest standard point of the assay, with a median of 500 pg/mL (34–11678). Fourteen of 27 HIV patients had CXCL13 concentrations above the lowest standard point of the assay. The median value of CXCL13 for the HIV patients was 10 pg/mL (3.9-498). All but one of the 39 controls had CXCL13 concentrations below the lowest standard point of the assay.

CSF CXCL13 levels between the LNB patients in the longitudinal and the cross-sectional part of the study differed (median 3727 pg/mL vs. 500 pg/mL) but the difference was not statistically significant (P = 0.065).

**Figure 4:**
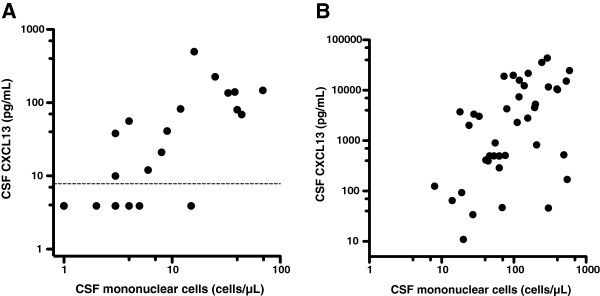
**Correlation between CSF mononuclear cells and CSF CXCL13 in HIV and Lyme neuroborreliosis.A**, Correlation between CSF mononuclear cells and CSF CXCL13 in untreated, asymptomatic HIV patients. Dotted line at the lowest standard point of the assay (7.8 pg/mL). (Spearman *r* = 0.81, P < 0.001). **B**, Correlation between CSF mononuclear cells and CSF CXCL13 in untreated Lyme neuroborreliosis (Spearman *r* = 0.55, P < 0.001). Lyme neuroborreliosis patients from the cross-sectional study and the pre-treatment part of the longitudinal study.

Correlations between CSF mononuclear cells and CXCL13 were calculated. For the HIV patients there was a significant correlation (Spearman *r* = 0.81, P < 0.001) (Figure 4A). For the LNB patients, the correlation was significant both in the cross-sectional study (Spearman *r* = 0.59, P = 0.016), and in the pre-treatment part of the longitudinal study (Spearman *r* = 0.40, P = 0.046). The combined analysis of the two groups of untreated LNB patients produced a Spearman *r* = 0.55, (P < 0.001) (Figure 4B).

For LNB patients, there was no significant correlation between the duration of neurological symptoms and CSF CXCL13 levels, either in the longitudinal study or in the cross-sectional study (data not shown).

**Figure 5:**
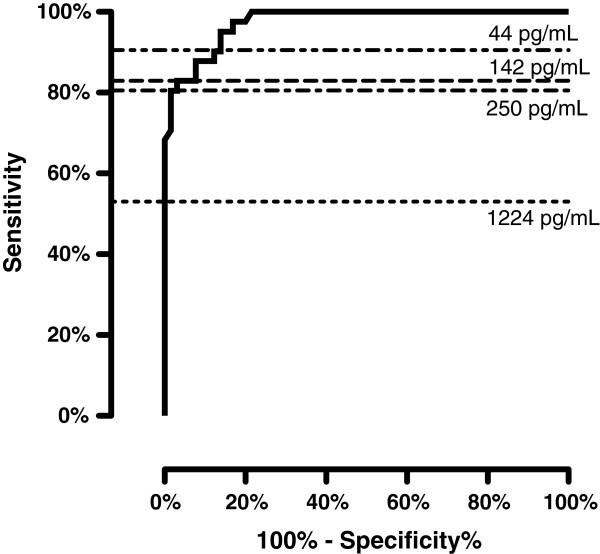
**ROC curve analysis of CSF CXCL13 levels to discriminate between Lyme neuroborreliosis patients and controls.** Lyme neuroborreliosis patients from the cross-sectional study and the pre-treatment part of the longitudinal study. Controls include asymptomatic HIV patients and study subjects with no CNS infectious or inflammatory disease. Horizontal lines show different suggested cut-off levels for CSF CXCL13 (Tjernberg et al. 142 pg/mL, van Burgel et al. 250 pg/mL, and Schmidt et al. 1,229 pg/mL). 61 pg/mL was the chosen cut-off in this study.

For the assessment of the diagnostic performance of CSF CXCL13, we combined the data on LNB patients from the cross-sectional study and the pre-treatment part of the longitudinal study and analysed them against the combined group of HIV patients and controls. A receiver operating characteristic (ROC) curve is shown in Figure 5. A CSF CXCL13 cut-off level of 61 pg/mL gives a sensitivity of 90% and a specificity of 88% for the diagnosis of LNB.

## Discussion

We confirm previous findings that CSF CXCL13 levels are highly elevated in untreated LNB patients. We also show that CSF CXCL13 concentrations decline after treatment with oral doxycycline and that the levels before and after treatment differ by about a hundredfold. This finding supports to the use of oral doxycycline for the treatment of LNB. Oral treatment has the advantages of being both cheaper and more convenient for the patient compared with intravenous treatment.

Does CSF CXCL13 concentrations add any valuable information to routine CSF analysis? Initially elevated CSF mononuclear cell count also declines significantly after treatment and the decline in levels of CSF mononuclear cells and CXCL13 are correlated, as shown in Figure 2. Even though the CSF cell count is unspecific, it has the advantage of being a rapidly available, easily performed test. As CSF mononuclear pleocytosis is one of the diagnostic criteria for LNB, its clinical usefulness is generally not assessed in studies of CSF CXCL13. However, especially when evaluating treatment effect and in diagnosing re-infection in patients with previous LNB, the extra value of CSF CXCL13 determination over CSF cell count is small, in our opinion. In spite of this, the lack of detectable *Bb*-specific antibody production can be seen in patients during the first few weeks of disease and, in a small number of patients, CSF pleocytosis may be absent for the first days of disease [17,18]. In this group of patients, analysis of CSF CXCL13 may provide additional information, as high CSF CXCL13 concentrations have been detected early in the infectious process, as shown in this and other studies [7]. A prospective study would be needed to confirm the clinical usefulness of CSF CXCL13 in this specific situation.

In the cross-sectional study, we chose to compare the levels of CSF CXCL13 LNB patients with patients with HIV infection for two reasons. Elevated serum CXCL13 levels have been described in HIV patients. Serum CXCL13 levels were higher in HIV patients with more advanced disease and correlated moderately with viral load [13]. In addition, HIV is known to cause a chronic, low-grade CNS infection in most asymptomatic patients, with a CSF picture resembling that of LNB, with mononuclear pleocytosis and oligoclonal IgG bands [19]. Elevated CSF CXCL13 levels have been described in HIV patients with clinical signs of CNS inflammation but not in asymptomatic HIV patients [10]. One inclusion criterion for HIV patients in this study was the absence of clinical signs of neurological disease. Nevertheless, 14/27 patients had CSF CXCL13 above the detection limit and, even though the difference at group level was significant, there was a clear overlap between the LNB patients and HIV patients (Figure 3). As highly elevated CSF CXCL13 concentrations has been reported in cryptococcosis and moderately elevated concentrations has been seen in African trypanosomiasis, the high concentrations reported here in asymptomatic HIV infection adds to the growing evidence that elevated CSF CXCL13 concentrations can not be regarded as specific for spirochetal CNS infections [10,20]. How this affects the diagnostic potential of CSF CXCL13 in LNB remains to be seen. Adding further to the problem is the fact that the most common clinical finding in LNB, peripheral facial palsy, is also reported among HIV patients [17,21].

The mechanism behind the previously reported elevated serum CXCL13 concentrations in HIV patients has not been determined. CXCL13 is known to be produced by macrophages, but the direct stimulation of macrophages and peripheral blood mononuclear cells by HIV-1 failed to induce CXCL13 production in one study [13]. Cagigi et al. demonstrated CXCL13 production in B cells from HIV patients but not in B cells from uninfected controls [22]. In this present study, we show a significant correlation between CSF mononuclear cells and CXCL13 (Figure 4A), but the correlation is not perfect and elevated CSF CXCL13 levels are seen in HIV patients without mononuclear pleocytosis. The exact mechanism behind the elevated serum and CSF CXCL13 concentrations seen in HIV patients remains to be elucidated.

It is difficult to compare CSF CXCL13 results from various studies. CSF CXCL13 has previously been related to CSF protein (and presented as CSF CXCL13/CSF protein, ng/g) to correlate with impaired blood brain barrier function and possible leakage into the CSF of CXCL13 [6]. The fact that CXCL13 levels in LNB patients are higher in CSF than in serum [23,24] indicates that CSF CXCL13 is produced intrathecally and absolute CSF CXCL13 values are therefore presented in our study and other recent studies [10,23,25]. We studied two separate groups of LNB patients. The median CSF CXCL13 values differed between the longitudinal study (3,727 pg/mL) and the cross-sectional study (500 pg/mL), although the difference did not reach statistical significance (P = 0.065). This cause of this difference is not clear. It could be a random effect, or it could related to the difference between the two groups in the CSF levels of mononuclear cells (median 118 cells/μL compared with median 58 cells/μL), as we show a significant correlation between CSF levels of mononuclear cells and CXCL13 in Figure 4B. The median value of 500 pg/mL in the cross-sectional study is lower than in other reports, which could be considered a weakness of this present study. However, two recent studies produced such widely differing mean and median values for CSF CXCL13 in LNB as 15,149 pg/mL (mean) and 1,183 pg/mL (median). The latter study used the same analytic kit as the one used in this study [10,23]. The differences between studies might reflect inter-centre variability or variability depending on the analytic kits used for measurements. Both are known problems for research-grade biomarker kits and kits for analyses for which certified reference methods and materials for kit calibration are lacking [26,27].

Previous studies of CSF CXCL13 in LNB have suggested optimal cut-off values, maximising sensitivity and specificity, where the level of CSF CXCL13 should be considered positive for LNB. Proposed cut-offs range from 142 pg/mL to 1,229 pg/mL (Tjernberg et al. 142 pg/mL, Schmidt et al. 1,229 pg/mL and van Burgel et al. 250 pg/mL) [10,23,25]. When these cut-off levels are applied to our material, they all give sensitivity below 83%, making them less useful. A cut-off value at such a low level as 61 pg/mL was needed to obtain acceptable sensitivity, when LNB was compared with a mixed population of non-infectious controls and asymptomatic HIV patients in our study (Figure 5). If CSF CXCL13 is to be used in clinical practice, the sensitivity and specificity must be estimated against relevant control groups, i.e. against populations with symptoms similar to those caused by LNB. The great difference between the suggested cut-off levels for CSF CXCL13 contrasts distinctly with the analysis of CSF mononuclear cells, which admittedly has a lower specificity for the diagnosis of LNB, but has a high sensitivity and also have a universally accepted cut-off level.

## Conclusions

We confirm previous reports of highly elevated CSF CXCL13 levels in LNB patients. CSF CXCL13 concentrations decline a hundredfold after treatment with oral doxycycline, which support the efficacy of this regimen. The same pattern is seen for CSF mononuclear cells. We show that CSF CXCL13 levels are also elevated in neurologically asymptomatic HIV patients and that the levels overlap those of LNB patients. CSF CXCL13 levels in LNB patients differ widely between studies. The diagnostic value of CSF CXCL13 in LNB remains to be established.

## Competing interests

NM has served on a scientific advisory board for Actelion Inc. LH has participated in a scientific advisory board for Pfizer, Astra Zeneca and Meda. The other authors report no competing interests.

## Authors’ contributions

DB, NM, HZ and LH came up with the idea for this study and participated in its conception and design. DB and LH drafted the manuscript. KB and UA participated in setting up the analyses. DB, ME, CW and LH recruited the study participants. NM and HZ made important revisions to the manuscript. All the authors read and approved the final manuscript.

## Pre-publication history

The pre-publication history for this paper can be accessed here:

http://www.biomedcentral.com/1471-2377/13/2/prepub
